# A joint genome-wide association study reveals candidate genes associated with egg production traits in chickens

**DOI:** 10.1016/j.psj.2026.107990

**Published:** 2026-10-17

**Authors:** Ming Fu, Jinsong Pi, Yaru Chen, Ailuan Pan, Hao Zhang, Tao Huang, Yan Wu

**Affiliations:** a Institute of Animal Sciences and Veterinary Medicine, Hubei Academy of Agricultural Sciences, Wuhan, 430064, China; b Key Laboratory of Animal Embryo Engineering and Molecular Breeding of Hubei Province, Wuhan, 430064, China

**Keywords:** Genome-wide association study, Selection signature, Egg production traits, Genetic parameters, Candidate genes

## Abstract

Egg production traits represent key economic determinants in the poultry industry, and unravelling their genetic architecture is essential for implementing genomic breeding in laying hens. Here, we performed a joint genome-wide association study (GWAS) with whole-genome sequencing data from 975 chickens (490 indigenous Jianghan chickens and 485 Rhode Island Red layers) to characterise the genetic basis of nine egg production traits: age at first egg (AFE), body weight at first egg (WFE), stage-specific egg number (EN) across the laying cycle, and maximum consecutive egg-laying days (MCD). Population genomic analyses revealed clear genetic differentiation between the two breeds. We further estimated moderate-to-high heritability for most assayed traits, as well as strong genetic correlations among stage-specific EN traits. A major shared quantitative trait locus (QTL) associated with WFE was mapped to chromosome 4 (GGA4), harboring key candidate genes *LDB2* and *PPARGC1A; IGF2BP1* on GGA27 was additionally identified as a promising candidate for this trait. For EN traits, three population-specific pleiotropic loci were detected, harboring candidate genes *ZNF592, DENND1B,* and *WDPCP* respectively. For MCD, *BMPR1A* and *MYCBP2* were prioritized as prominent candidate genes in the two breeds. Candidate genes from selection signature analysis (SSA) were enriched in hormonal response and energy metabolism pathways, whereas GWAS-identified candidates were mainly associated with follicular development, calcium signaling, and apoptosis regulation. Taken together, our results reveal the layered genetic architecture of egg production traits in indigenous and commercial layer populations. The identified SNPs and candidate genes will provide molecular markers to support genetic improvement of laying performance in chicken breeding.

## Introduction

Egg production is one of the most important economic traits in laying hens, which directly determines the productivity and economic efficiency of the poultry industry (Zhang et al., 2026). Egg production can be evaluated by indicators such as age at first egg (AFE), egg number (EN), and maximum consecutive egg laying days (MCD). Among the evaluated traits, egg number (EN) is a key production indicator and is closely related to egg production capacity (Ma et al., 2024; Huang et al., 2025). Improving egg production traits is a major objective in layer breeding. However, as these traits typically exhibit low heritability, are genetically complex, and are controlled by multiple genes, the genetic progress achieved through conventional breeding approaches remains limited (Wolc et al., 2011; El-Sabrout et al., 2022; Büttgen et al., 2025). Therefore, excavating functional genes and molecular markers responsible for egg-laying variation is essential to carry out genomic breeding in chickens.

QTL mapping and genome-wide association study (GWAS) are extensively utilized to identify molecular markers linked to complex economic traits in chickens (Tan et al., 2025). Up to now, the Chicken QTL database has recorded 947 QTLs related to EN traits and 168 QTLs linked to AFE (https://www.animalgenome.org/cgi-bin/QTLdb/GG/index). Existing candidate gene research predominantly focuses on the conserved hypothalamic-pituitary-gonadal (HPG) axis, which governs follicle development and ovulation (Du et al., 2020; Wadood et al., 2025). Key reproductive genes including gonadotropin-releasing hormone (*GnRH*), gonadotropin-inhibitory hormone (*GnIH*), neuropeptide Y (*NPY*), follicle-stimulating hormone receptor (*FSHR*), and prolactin receptor (*PRLR*) have been repeatedly identified as functional regulators of laying performance in independent population cohorts (Hu et al., 2015; Hanlon et al., 2021). Beyond these classic HPG axis genes, recent high-resolution GWAS have identified dozens of novel genomic regions and unreported candidate genes significantly associated with egg-laying capacity across diverse chicken populations (Christoffels et al., 2011; Goto et al., 2011; Yuan et al., 2017).

To uncover candidate genes and genetic variants governing egg production traits, we performed a joint GWAS using whole-genome sequencing on two populations, including 490 Chinese indigenous Jianghan chickens and 485 commercial Rhode Island Red chickens. As a renowned local chicken breed of China, Jianghan chicken possesses prominent advantages including robust disease resistance, broad environmental adaptability, and high-quality meat and egg products. In contrast, the Rhode Island Red is a classic commercial chicken breed, which has undergone long‑term directional selection for egg‑laying performance and formed specialized layer lines (Chen et al., 2024; Kochetova et al., 2026). Through comparative analysis of genomic variations between the indigenous Jianghan chickens and the commercial Rhode Island Red chickens, this study aimed to prioritize key genomic regions and functional genes closely associated with egg production traits. The findings will provide a valuable molecular basis and theoretical support for genetic improvement of laying performance in chicken breeding.

## Materials and methods

### Animals and phenotypic measurement

In this study, a total of 975 chickens, including 490 Jianghan chickens (A line) and 485 Rhode Island Red chickens (H line) (Fig. 2A), were collected from the Jinshui experimental station (Wuhan, Hubei Province, China). Jianghan chicken is an indigenous breed from Hubei Province, China. Rhode Island Red chicken is a specialized brown-egg laying breed. The experimental chickens were reared in stacked single cages with routine feeding and husbandry practices. Data for egg production traits were collected every day starting from the first egg to 40 weeks of age. The phenotyping measurement includes four traits: age at first egg (AFE), body weight at first egg (WFE), egg number (EN), and maximum consecutive egg laying days (MCD). According to the distinct patterns of laying curves for populations A and H (Fig. S1), the laying cycle was separated into three critical stages: AFE to peak laying point (egg number at 30 weeks of age) (EN30), declining laying point (egg number at 35 weeks of age) (EN35) and totle laying point (egg number at 40 weeks of age) (EN40). In this study, EN1 represented the egg number recorded from 36 to 40 weeks of age, EN2 stood for egg production from 31 to 35 weeks of age, and EN3 referred to the cumulative egg yield across the 30–40 week laying period.

We obtained blood samples from the brachial vein of every chicken in accordance with the standard sampling protocol defined in this breeding project. The animal experiment scheme received ethical approval from the Animal Care and Use Committee of the Institute of Animal Science and Veterinary Medicine, Hubei Academy of Agricultural Sciences. No invasive surgical procedures were performed in this study. All procedures were designed to minimize animal stress.

### DNA extraction and whole genome sequencing

We extracted genomic DNA from blood specimens by applying the HiPure Universal DNA Kit (Magen Biotechnology, China) as instructed by the kit supplier. A NanoDrop 2000 Spectrophotometer (Thermo Scientific, Waltham, MA, USA) was used to detect DNA concentration and purity. The recovered DNA was kept at −80 °C to prepare for downstream analyses. A total of 975 chickens were genotyped via whole-genome sequencing conducted by Wuhan Yingzi Gene Technology Co., Ltd (Wuhan, China). The sequencing platform produced high-quality paired-end reads of 150 bp, achieving 10 × coverage for each sample.

### Genotyping and quality control

All the clean reads were aligned to the chicken reference (GRCg6a, GCA_000002315.5) using Burrow-Wheeler Alignment (BWA) software v0.1.17 (Li and Durbin, 2009). The obtained reads were realigned using GATK software v4.1.8.1 (McKenna et al., 2010). We conducted SNP filtering analyses using the PLINK v1.90 (Purcell et al., 2007) with parameters as follows: plink -geno 0.05 -maf 0.01 -hwe 0.000001. We utilized the Beagle v5.0 algorithm (Browning and Browning, 2007) to impute missing genotypes of the remaining SNPs. Ultimately, a total of 7,346,595 valid SNPs were acquired from the 975 sequenced chicken samples.

### Population structure analysis

To explore the genetic relationship between the populations A and H, principal component analysis (PCA) was conducted using PLINK v1.90. Additionally, the population structure of these chicken populations was inferred using ADMIXTURE software v1.30 (Alexander and Lange, 2011), as described by Shringarpure (Shringarpure et al., 2016). Linkage disequilibrium (LD) decay analysis was performed using PopLDdecay software v3.40 (Zhang et al., 2019), establishing a proper cutoff interval to retrieve candidate genetic variants.

### Selection signature analysis

We used VCFtools software v0.1.15 (Danecek et al., 2011) to estimate genome-wide Fst values and Pi ratios of group A versus group H, with sliding windows of 50 kb and 10 kb increments. Log2-transformation was applied to the Pi ratios (A/H). Those windows ranked among the upper 5% for both metrics were identified as candidate positive selection regions, which were then subjected to gene annotation and functional enrichment.

### Genetic parameter estimation

Genetic parameters including heritability and pairwise genetic correlations were calculated via the AI-REML algorithm. This analytical approach, embedded in the GCTA v1.94 software package, was applied in accordance with previously established methodologies (Yang et al., 2011).

### Genome-wide association study

GWAS was performed by mixed linear model (MLM) in the GEMMA software v0.98.5 (Zhou and Stephens, 2012). This approach was adopted to quantify the fraction of phenotypic variance attributable to candidate SNPs and haplotypes. For the joint GWAS in the combined population AH, breed was added as a fixed-effect covariate to control for population stratification, in addition to the top three principal components. Bonferroni correction was adopted to correct for multiple statistical tests. The calculated thresholds for suggestive and genome-wide significance were 1.36E-07 (1/7346595) and 6.81E-09 (0.05/7346595), respectively. The CMplot R package (available at https://github.com/YinLiLin/CMplot) was utilized in R 4.2.3 to visualize the GWAS outcomes, encompassing Manhattan plots and quantile-quantile (QQ) plots.

### Analysis of linkage disequilibrium and screening of candidate genes

Linkage disequilibrium (LD) analysis was performed by LDBlockShow software v1.40 (Dong et al., 2021). Gene annotation data retrieved from Ensembl genome browser 116 (http://asia.ensembl.org/index.html) was used to screen candidate genes flanking the significant SNPs and those located within target genomic regions. To explore the potential biological functions and signaling pathways of the screened genes, Gene Ontology (GO) functional annotation and Kyoto Encyclopedia of Genes and Genomes (KEGG) pathway enrichment analyses were carried out via the online OmicShare Tools platform (https://www.omicshare.com).

## Results

### Phenotypic traits summary

Descriptive statistics for egg production traits and pairwise phenotypic correlations among traits are provided in Table 1 and Fig. 1, respectively. Marked phenotypic divergence in laying performance was detected between populations A and H. All evaluated laying traits differed significantly between the two populations (*P* < 0.0001), with the sole exception of EN35. Notably, AFE showed a strong, highly significant negative correlation with EN30, EN35, and EN40.

**Table 1:** Descriptive statistics for egg production traits.

Traits	Breed	N	Min	Max	Mean	SD	CV (%)
AFE	A	490	109	177	145.9	12.9	8.8
	H	485	129	174	153.2	9.5	6.2
	AH	975	109	177	149.5	11.9	8.0
WFE	A	490	760	1540	1102.1	127.5	11.6
	H	485	950	1690	1304.8	134.4	10.3
	AH	975	760	1690	1203.0	165.6	13.8
EN40	A	490	54	153	104.2	17.6	16.9
	H	485	68	147	112.2	13.0	11.6
	AH	975	54	153	108.2	16.0	14.8
EN35	A	490	47	120	81.4	13.9	17.1
	H	485	46	113	82.0	11.2	13.7
	AH	975	46	120	81.7	12.6	15.4
EN30	A	490	28	92	54.8	12.1	22.1
	H	485	24	79	51.0	10.4	20.4
	AH	975	24	92	52.9	11.4	21.6
EN1	A	490	0	35	22.7	6.8	30.0
	H	485	0	35	30.2	4.6	15.2
	AH	975	0	35	26.4	6.9	26.1
EN2	A	490	8	36	26.7	4.9	18.4
	H	485	13	37	31.0	3.2	10.3
	AH	975	8	37	28.8	4.7	16.3
EN3	A	490	13	69	49.4	10.5	21.3
	H	485	22	71	61.2	6.8	11.1
	AH	975	13	71	55.3	10.6	19.2
MCD	A	490	4	89	18.1	13.7	75.7
	H	485	6	119	33.7	20.9	62.0
	AH	975	4	119	25.9	19.3	74.5

AH: combined population of A and H; AFE: age at first egg; WFE: body weight at first egg; EN40, EN35, EN30: egg number at 40, 35, and 30 weeks of age, respectively; EN1: egg number from 36 to 40 week; EN2: egg number from 31 to 35 weeks; EN3: egg number from 31 to 40 weeks; MCD: maximum consecutive egg laying days.

**Fig. 1:**
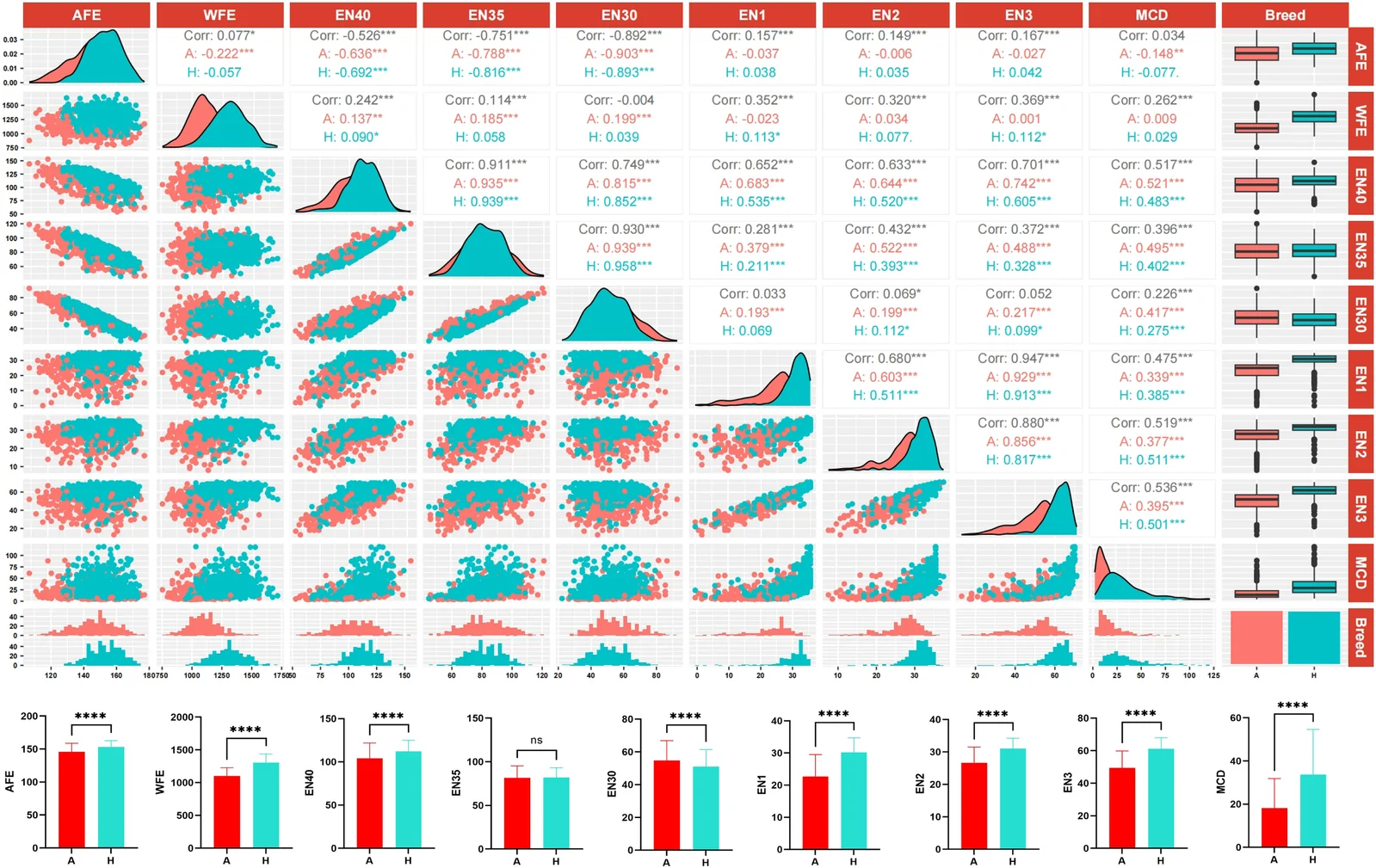
Phenotypic data analysis among populations A, H, and AH. * *P* < 0.05, ** *P* < 0.01, *** *P* < 0.001, **** *P* < 0.0001.

### Population genetics analysis and selection signature analysis

After whole-genome sequencing followed by strict quality filtering procedures, a final set of 7346595 high-quality SNPs was retained for subsequent analyses. The SNP density plot was shown in Fig. 2B. Principal component analysis (PCA) based on the first three principal components revealed clear genetic stratification between populations A and H (Fig. 2C). Linkage disequilibrium (LD) decay analysis showed that population A exhibited a faster LD decay rate than population H (Fig. 2D). The cross-validation error bar plot for admixture analysis is presented in Fig. 2E, and the optimal clustering parameter was determined at K = 2. Under this optimal genetic grouping, populations A and H were distinctly assigned into two independent clusters. Integrating the phenotypic divergence, stratified population structure, and differential LD decay patterns, our results collectively demonstrated significant genetic differentiation between populations A and H.

**Fig. 2:**
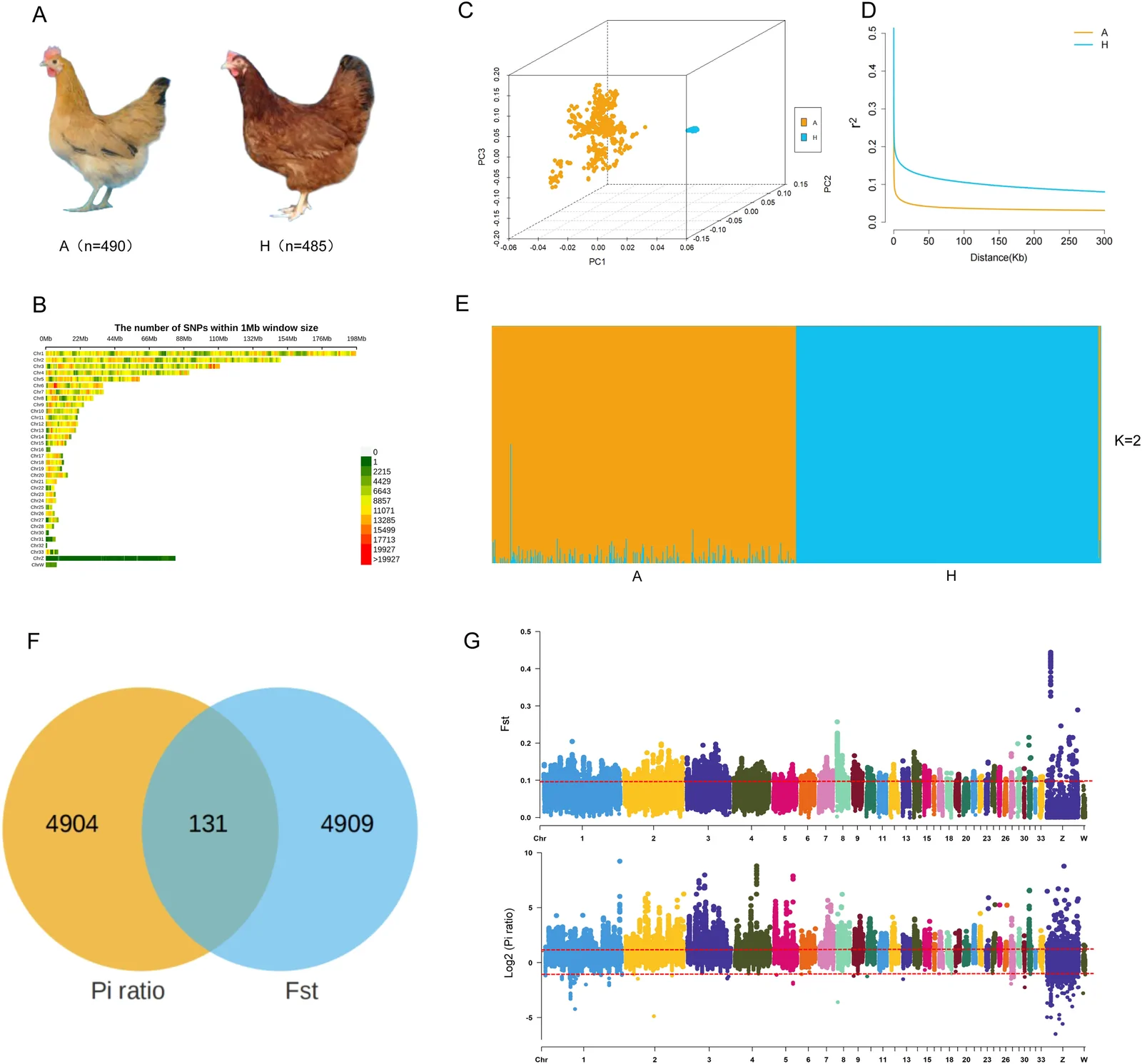
Genomic analysis of populations A and H. (A) Population sizes of A and H. (B) SNP density plot. (C) Principal component analysis. (D) Linkage disequilibrium (LD) decay analysis. (E) Admixture analysis. (F-G) Selection signature analysis (SSA) by Fst and Pi ratio.

To screen genetically differentiated genomic regions, we performed selection signature analysis (SSA) using Fst and Pi ratio. Fst and Pi ratio analyses detected 5040 and 5035 candidate genomic regions separately (Fig. 2F, G, Tables S1–S2), with 131 overlapping regions shared by both datasets. Annotation analysis of these overlapping regions identified a total of 52 candidate genes (Table S3).

### Estimation of genetic parameters

Estimates of SNP-based heritability and genetic correlations for AFE, WFE, ENs, and MCD are listed in Table S4. The heritability estimates of the nine traits ranged from 0.216 to 0.558 in population A and from 0.047 to 0.519 in population H. Notably, the heritability values of egg production traits were generally higher in population A than those in population H. The heritability of WFE was 0.577 and 0.482 in population A and population H, respectively, indicating that WFE belongs to a trait with moderate-to-high heritability. Furthermore, significantly positive genetic correlations were detected among different EN indicators. In contrast, AFE exhibited negative genetic correlations with EN30, EN35, and EN40. This pattern was also reflected at the phenotypic level, whereby AFE was negatively correlated with the above traits.

### Summary of GWAS

We performed GWAS to identify SNPs associated with AFE, WFE, ENs, and MCD in population A, population H, and the combined population AH, respectively. Manhattan and QQ plots for populations A and H are provided in Figs. S2–S5, whereas the corresponding plots for the combined population AH are displayed in Fig. 3 and Fig. S6. In total, 554 genome-wide significant SNPs and 561 suggestive SNPs were detected for these traits. Functional annotation of these significant SNPs identified 120 candidate genes (Table S5). The top significant SNP for each trait across population A, population H, and the combined population AH is listed in Table 2.

**Fig. 3:**
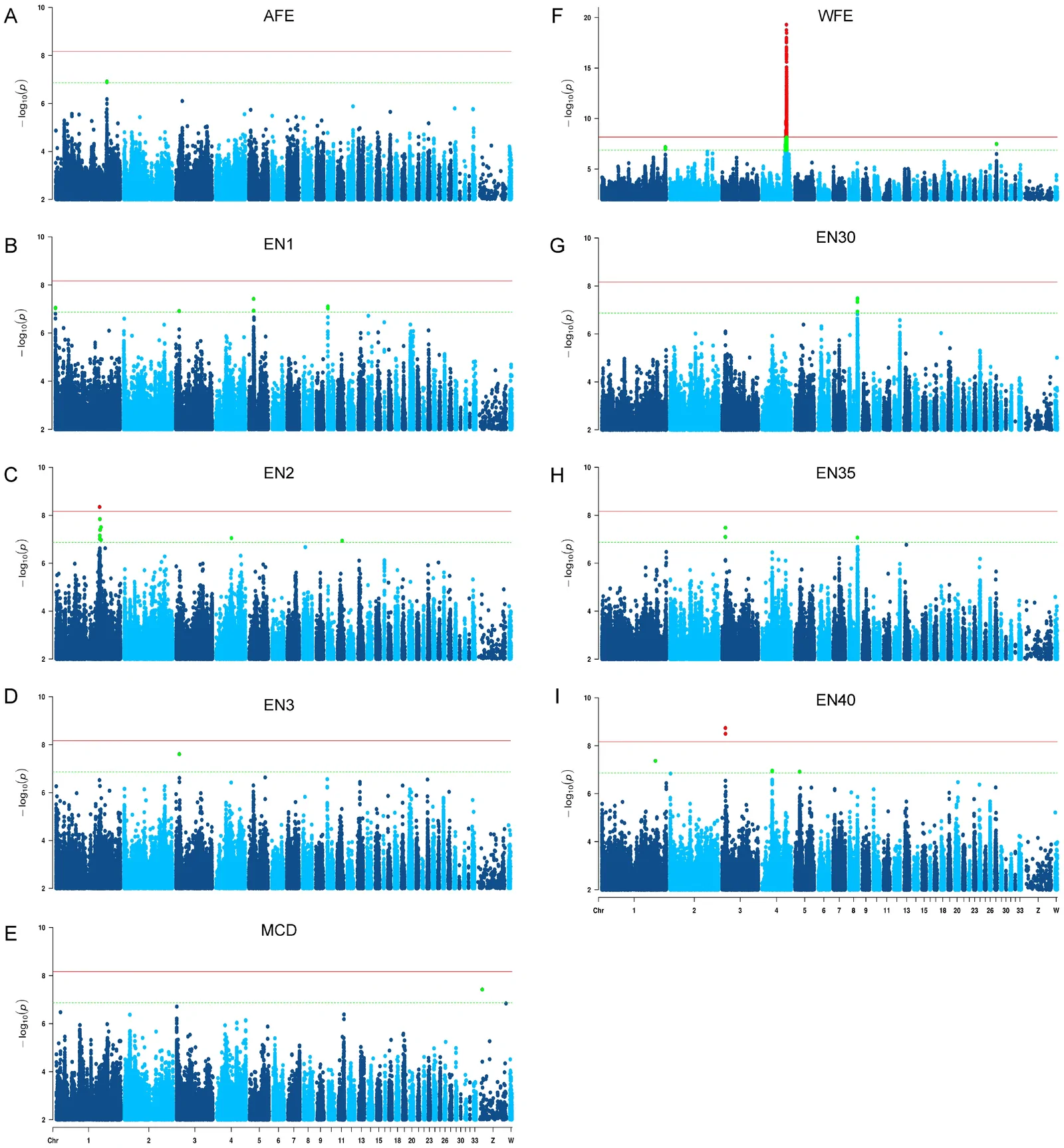
Manhattan plot of SNP-based genome-wide asscoaition signals for egg production traits in combined population AH. The solid red and green lines represent the genome-wide significance threshold and suggestive threshold, respectively.

**Table 2:** The most significant SNPs associated with target traits in population A, population H, and combined AH population.

Traits	Breed	Chr	SNP	MAF^a^	P-value	PVE^b^	Candidate Gene
AFE	H	1	1:155430421	0.078	7.90E-08	2.96	*EDNRB, SLAIN1, MYCBP2*
	AH	1	1:156901469	0.219	1.22E-07	2.83	*TBC1D4, KLF12*
WFE	A	4	4:77325222	0.469	1.60E-09	3.74	*RAB28*
	H	4	4:76004672	0.154	7.67E-16	6.67	*QDPR*
	AH	4	4:75727946	0.375	5.06E-20	8.26	*LCORL, LDB2*
EN1	A	20	20:13081372	0.362	2.82E-08	3.16	*SALL4, ATP9A, NFATC2*
	H	15	15:5574864	0.01	1.29E-11	4.71	*ARPC3, GPN3, DENR*
	AH	5	5:14329494	0.068	3.83E-08	3.06	*CTSD*
EN2	A	1	1:132966607	0.186	1.22E-08	3.33	*GABRB3*
	H	3	3:76425359	0.047	2.51E-08	3.19	*SLC35A1, ORC3*
	AH	1	1:133187233	0.013	4.50E-09	3.47	*UBE3A*
EN3	A	10	10:1445323	0.312	3.31E-08	3.13	*ZNF592, PCASP3, ALPK3*
	H	15	15:5574864	0.01	2.50E-10	4.11	*ARPC3, GPN3, DENR*
	AH	3	3:10090166	0.012	2.48E-08	3.14	*WDPCP, MDH1, UGP2, VPS54*
EN30	H	1	1:3889188	0.013	4.41E-10	4.00	*IL2RA, RBM17*
	AH	8	8:27200096	0.018	3.24E-08	3.09	*NFIA*
EN35	H	18	18:504917	0.018	3.61E-09	3.58	*MYH1B*
	AH	3	3:10332983	0.022	3.32E-08	3.08	*LGALSL, PELI3*
EN40	A	3	3:10090166	0.024	5.09E-08	3.05	*WDPCP, MDH1, UGP2, VPS54*
	H	8	8:2817254	0.027	3.93E-08	3.10	*ZBTB41, LHX9, DENND1B*
	AH	3	3:10090166	0.012	1.82E-09	3.64	*WDPCP, MDH1, UGP2, VPS54*
MCD	A	6	6:3271811	0.013	1.74E-09	3.72	*BMPR1A*
	H	1	1:155592877	0.219	9.62E-08	2.92	*MYCBP2*
	AH	Z	Z:8389318	0.02	3.80E-08	3.06	*IL11RA, CCL19*

a MAF indicates the minor allele frequency of first listed marker.

b PVE percentage of phenotypic variance explained by each SNP.

### Screening of significant SNPs associated with WFE

Manhattan and QQ plots of WFE for population A, population H, and the combined population AH are shown in Fig. 4A and Fig. S7. GWAS of WFE revealed 47 associated SNPs (7 genome-wide significant) in population A, 431 associated SNPs (205 genome-wide significant) in population H, and 518 associated SNPs (330 genome-wide significant) in the combined population AH. These associated SNPs were distributed on chromosomes 1, 4, 19, 27, and 33 (Table S5). Among these associated SNPs, 10 SNPs were commonly identified in three populations (Fig. 4B). These SNPs exhibited strong linkage disequilibrium, among which the peak SNP was chr4:75727946 (Fig. 4C). We further statistically analyzed the phenotypic distribution corresponding to this variant in the three populations, and found that individuals carrying the GG genotype possessed significantly higher phenotypic values than those with AG and AA genotypes (Fig. 4D, E, F).

**Fig. 4:**
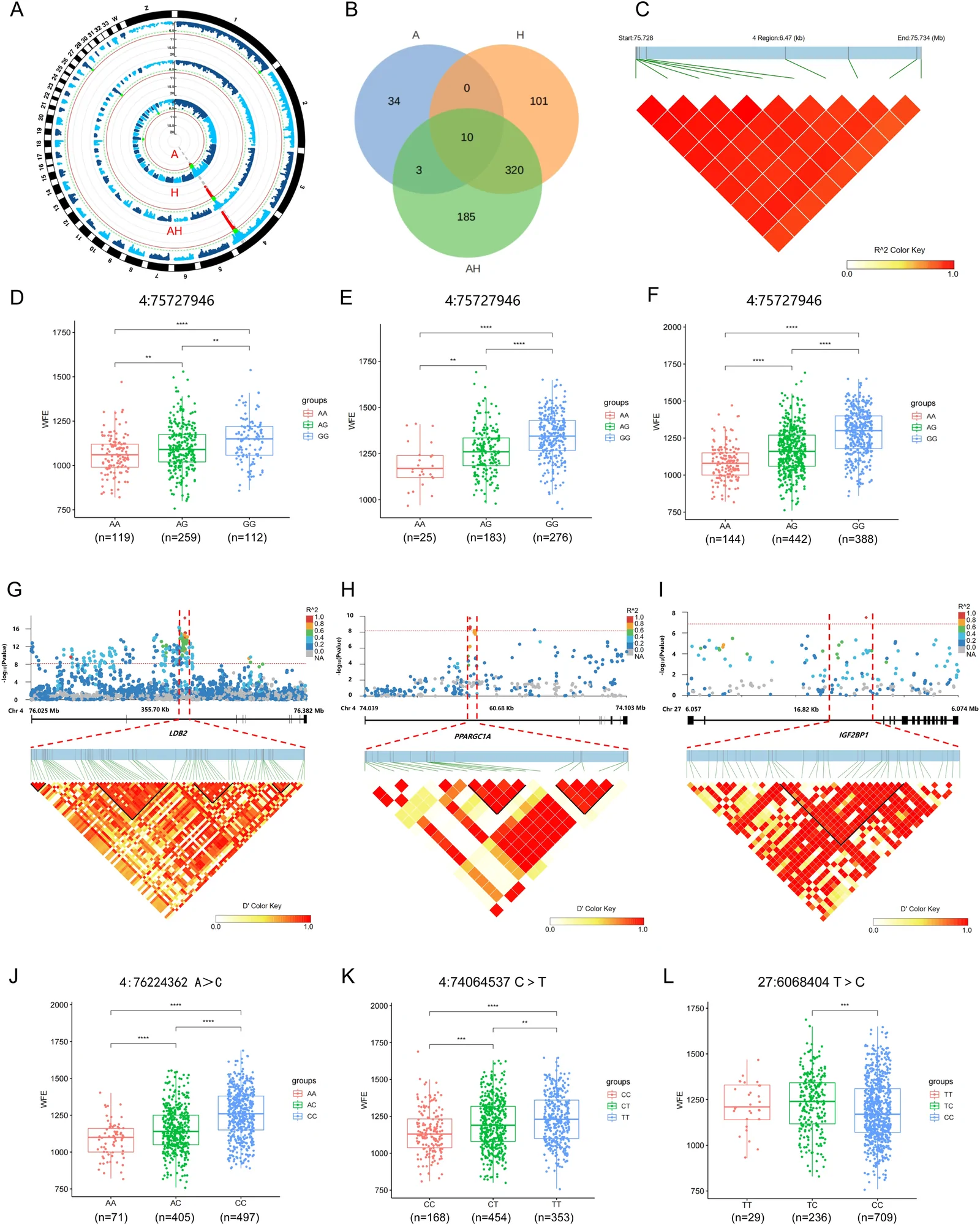
Identification of genetic loci that associate to WFE. (A) GWAS manhattan plot for WFE in population A, population H, and the combined population AH. (B) Venn diagram illustrating the overlap of significant SNPs associated with WFE identified by GWAS in populations A, H, and AH. (C) LD analysis for the overlap SNPs. (D-F) Phenotypic differences of WFE in populations A, H, and AH stratified by genotypes of the most significant shared SNP. (G-I) LD analysis surrounding the peak on candidate genes (*LDB2, PPARGC1A, IGF2BP1*). (J-L) Phenotypic differences of WFE in population AH stratified by genotypes of the most significant SNP. ** *P* < 0.01, *** *P* < 0.001, **** *P* < 0.0001.

Furthermore, functional annotation of the significant SNPs identified three key candidate genes, LIM domain binding 2 (***LDB2***), peroxisome proliferator-activated receptor gamma coactivator 1 alpha (***PPARGC1A***), and insulin-like growth factor 2 mRNA-binding protein 1 (***IGF2BP1***), on chromosomes 4 and 27. A total of four independent LD blocks covering 77 SNPs were pinpointed in the genomic region of the *LDB2* gene. The top associated SNP at chr4:76224362 served as the peak variant in this interval, with robust genotype-phenotype association signals detected in the locus (Fig. 4G, J). Within the *PPARGC1A* gene region (including 19 SNPs), two distinct LD blocks were identified, the peak SNP in this region was located at chr4:74064537, which exhibited significant genotype-phenotype associations (Fig. 4H, K). Within the *IGF2BP1* gene region (including 39 SNPs), a prominent LD block was identified, the peak SNP in this region was located at chr27:6068404, which exhibited significant genotype-phenotype associations (Fig. 4I, L).

### Screening of significant SNPs associated with ENs

Genome-wide association studies for egg number traits were separately performed in population A, population H, and combined population AH. A total of 13 associated SNPs were detected in population A (Table S5), among which one SNP overlapped across three egg number phenotypes (EN40, EN1, and EN3) (Fig. 5A). This shared variant was located in the intronic region of zinc finger protein 592 ‌(***ZNF592***) on chromosome 10. LD structure analysis characterized one discrete block spanning 24 SNPs in the *ZNF592* gene region. The variant chr10:1445323 was recognized as the peak SNP of this locus, with strong genotype-phenotype association signals observed in this genomic interval (Fig. 5E, H).

**Fig. 5:**
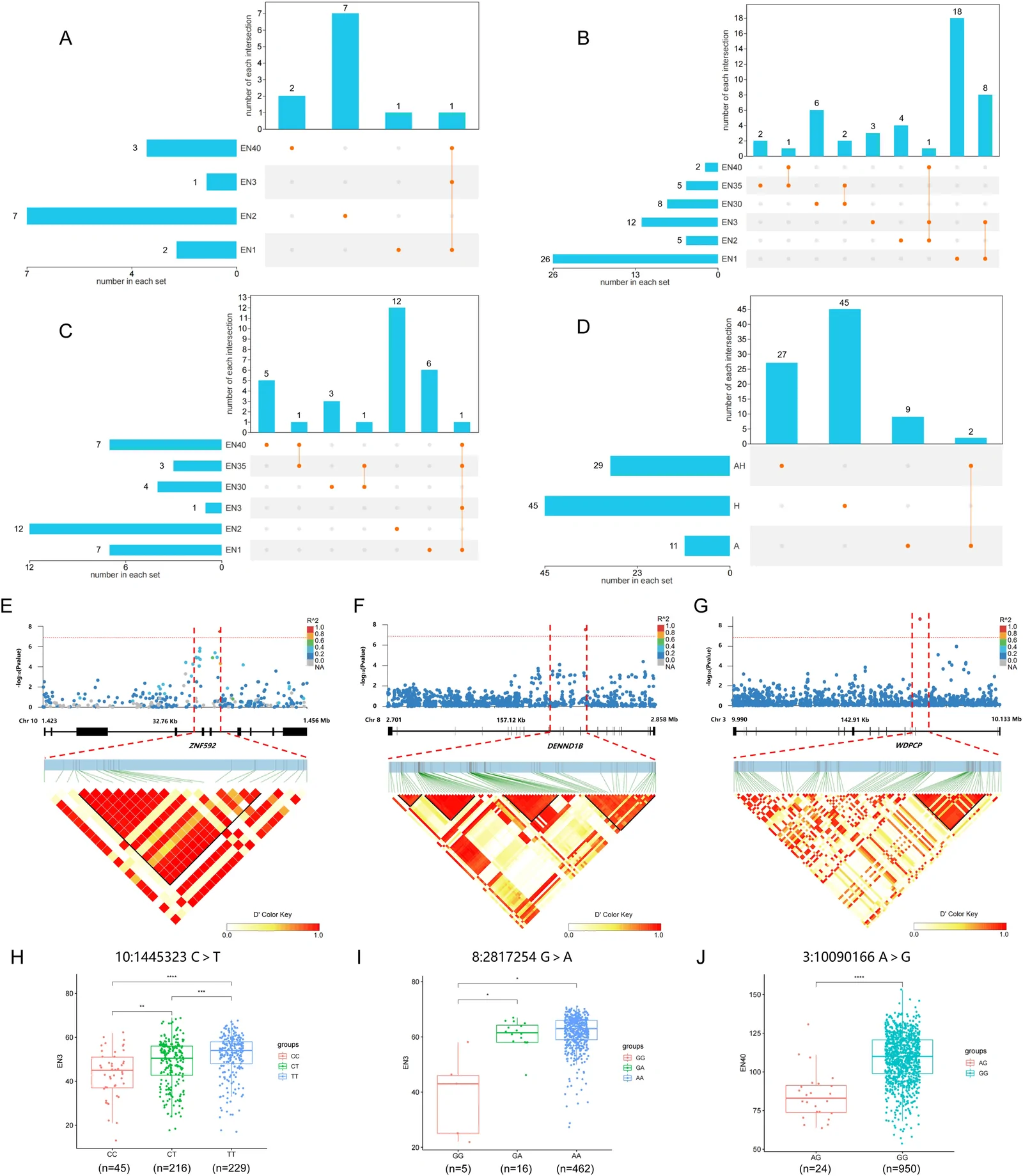
Identification of genetic loci that associate to ENs. (A-D) Upset plot quantifying the overlap of significant SNPs associated with egg number traits derived from GWAS in population A, population H, and combined population AH. (E-G) LD analysis surrounding the peak on candidate genes (*ZNF592, DENND1B, WDPCP*). (J-L) Phenotypic differences of ENs in population AH stratified by genotypes of the most significant SNP. * *P* < 0.05, ** *P* < 0.01, *** *P* < 0.001, **** *P* < 0.0001.

In population H, 58 associated SNPs associated with egg production were identified (Table S5), including one pleiotropic SNP simultaneously associated with EN40, EN2, and EN3 (Fig. 5B). This variant resided within the intron of ‌DENN domain containing 1B (***DENND1B***) on chromosome 8. Within the *DENND1B* gene region (including 91 SNPs), four distinct LD blocks were identified, the peak SNP in this region was located at chr8:2817254, which exhibited significant genotype-phenotype associations (Fig. 5F, I).

For the combined population AH, 34 significant SNPs were obtained (Table S5), and one pleiotropic SNP was shared by EN40, EN35, EN1, and EN3 (Fig. 5C), which mapped to the intronic region of WD repeat containing planar cell polarity effector (***WDPCP***) on chromosome 3. Analysis of the *WDPCP* genomic region uncovered one independent LD block composed of 91 SNPs. The core peak variant chr3:10090166 presented the strongest genotype-phenotype association within this locus (Fig. 5G, J).

An Upset plot was used to quantify the overlaps of significant SNPs across the three populations.Two shared SNPs were found between population A and combined population AH. Importantly, one shared SNP corresponded exactly to the multi-phenotype pleiotropic locus discovered in AH. No overlapping significant SNPs were detected between A and H, H and AH, or across all three populations (Fig. 5D).

### Screening of significant SNPs associated with MCD

For the MCD trait, only one genome-wide significant SNP was exclusively detected in population A, which mapped to the intronic region of bone morphogenetic protein receptor type 1A (***BMPR1A***). No genome-wide significant SNPs associated with MCD were discovered in population H and combined population AH (Table S5).

At the suggestive association level, seven suggestive SNPs were obtained in population A. The single suggestive SNP identified in population H was located within the intron of MYC binding protein 2 (***MYCBP2***), while combined population AH harbored one suggestive SNP for MCD (Table S5). Further characterization of the *MYCBP2* gene region detected four discrete LD blocks. Among these loci, the peak SNP situated at chr1:155592877 displayed robust genotype-phenotype association signals (Fig. 6A, B).

**Fig. 6:**
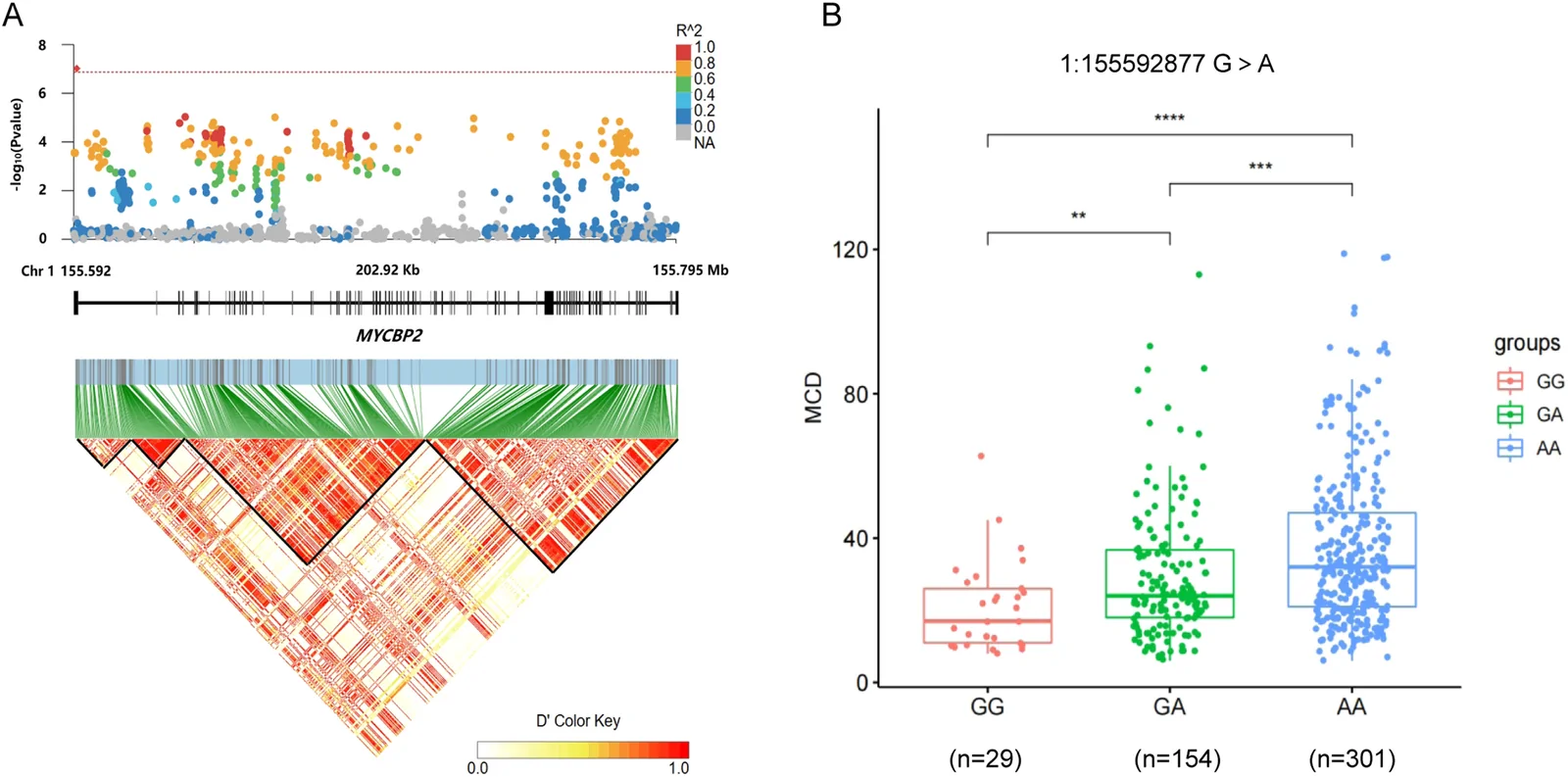
Identification of genetic loci that associate to MCD. ** *P* < 0.01, *** *P* < 0.001, **** *P* < 0.0001.

### Functional enrichment analyses of candidate genes

Firstly, we compared the candidate genes identified by SSA and GWAS with previously reported egg production trait-related genes in the chicken QTLdb (Fig. 7A, Table S6). Two genes, *LSAMP* and *FARSB* (Ding et al., 2022; Lei et al., 2024), overlapped between the SSA-derived candidates and QTLdb, while *NR3C2* (Xu et al., 2015) was shared between GWAS candidates and documented QTLdb genes. Of note, no candidate genes were commonly detected by SSA and GWAS in the present study.

**Fig. 7:**
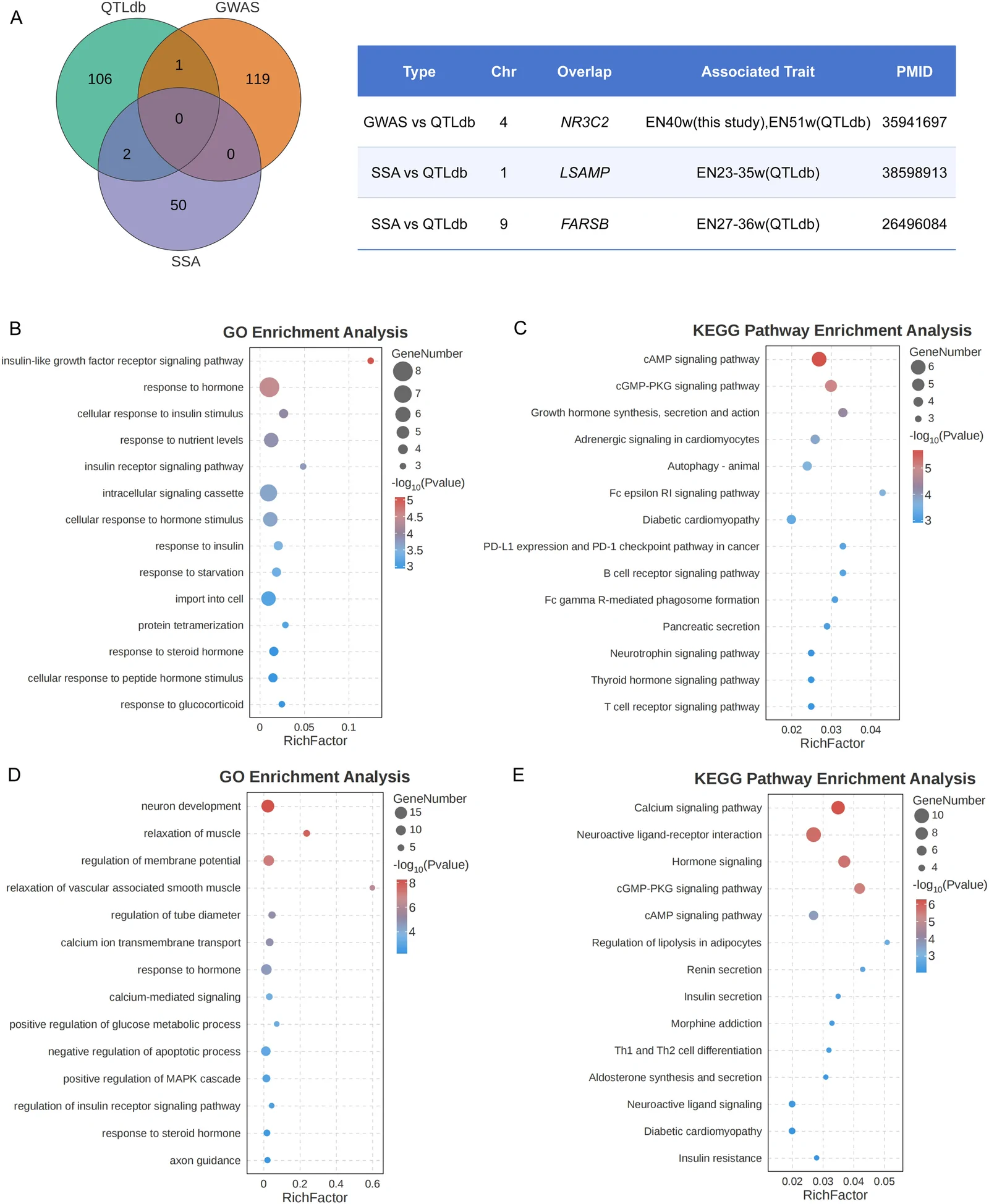
Functional enrichment analysis of SSA and GWAS candidate genes. (A) Venn plot showing overlaps of egg-production-related candidate genes among SSA, GWAS and chicken QTLdb. (B, C) GO and KEGG annotation analysis for candidate genes of SSA. (D, E) GO and KEGG annotation analysis for candidate genes of GWAS.

Subsequently, GO enrichment and KEGG pathway enrichment analyses were independently conducted for candidate genes derived from SSA and GWAS, to annotate biological functions and enriched pathways. For the SSA-derived gene set, a total of 14 biological process terms were significantly enriched, including the insulin-like growth factor receptor signaling pathway, response to hormone, and response to nutrient levels (Fig. 7B, Table S7). KEGG pathway analysis further revealed significant enrichment in multiple reproductive and metabolic signaling pathways, such as the cAMP signaling pathway, cGMP-PKG signaling pathway, and growth hormone synthesis, secretion and action pathway (Fig. 7C, Table S8).

For the GWAS candidate genes, 14 significantly enriched biological process terms were also identified, mainly involving neuron development, muscle relaxation, and negative regulation of the apoptotic process (Fig. 7D, Table S9). Correspondingly, KEGG pathway enrichment results demonstrated that the screened genes were markedly enriched in pivotal biological pathways, mainly covering calcium signaling, neuroactive ligand-receptor interaction, and hormone signaling transduction processes (Fig. 7E, Table S10).

## Discussion

Egg production represents a primary economic trait for layer breeding, and elucidating its genetic basis is essential for genomic‑assisted improvement. In the present study, a joint GWAS was performed using two genetically divergent populations: indigenous Jianghan chickens (A line) and commercial Rhode Island Red chickens (H line). This design expands the pool of segregating variants and increases statistical power to capture both shared and population‑specific QTL underlying egg production traits. Given the substantial genetic differentiation between the two lines, breed was incorporated as a fixed‑effect covariate alongside PCA components to mitigate false‑positive associations caused by population stratification. Such a framework also supports integrated interpretation of GWAS and selection signature results to dissect the genetic basis of phenotypic divergence between indigenous and commercial layer chickens.

### Phenotypic divergence and population genetic structure

We detected substantial phenotypic divergence in laying performance between Jianghan and Rhode Island Red chickens. Rhode Island Red hens had greater WFE, ENs, and MCD, while Jianghan chickens attained AFE at a younger age. These results illustrate that commercial layers, although reaching sexual maturity later than the indigenous Jianghan chickens, possess better laying persistence and more consistent egg production throughout the late laying phase. AFE showed moderate-to-high heritability (0.497 in A and 0.519 in H), and exhibited strong negative phenotypic and genetic correlations with EN traits in different weeks of age, which is consistent with previous studies (Chen et al., 2024; Liu et al., 2025; Zhang et al., 2026). Accordingly, AFE was included as a covariate in the mixed‑linear model for GWAS of EN traits to remove its confounding effect on egg number phenotypes. Heritability estimates were generally higher in Jianghan chickens, implying greater genetic improvement potential retained in this indigenous breed.

Population genomic analyses revealed pronounced genetic differentiation between the two breeds. Both principal component analysis (PCA) and ADMIXTURE analysis resolved the two populations into distinct, non-overlapping genetic clusters. Jianghan chickens exhibited more rapid linkage disequilibrium (LD) decay, a hallmark of indigenous breeds that have not undergone intensive artificial selection. This strong genetic differentiation provides a sound foundation for selection signature scans, and also accounts for the finding that most GWAS associations were population-specific rather than shared across the two populations. These divergent LD patterns reflect distinct breeding histories and selection intensities between the lines, and likely have shaped the contrasting genetic architectures underlying egg production traits.

### Major candidate genes associated with WFE

WFE is tightly correlated with egg number traits and exerts a direct effect on subsequent egg‑laying performance (Yang et al., 2024). In this study, we detected a strong and shared association signal on GGA4 across all three populations (A, H, and AH), with the peak SNP at chr4:75727946. Ten shared significant SNPs in this region formed a strong LD block, and individuals with the GG genotype consistently had significantly higher WFE in all three populations, indicating that this locus is a stable major QTL. Furthermore, three pivotal candidate genes, including *LDB2, PPARGC1A,* and *IGF2BP1*, were uncovered on GGA4 and GGA27 by LD analysis.

*LDB2* has been repeatedly associated with growth and body weight traits in chickens (Wei et al., 2020; Dou et al., 2022; Fu et al., 2023), and it participates in transcriptional regulation of cell proliferation and tissue development. *PPARGC1A* is a core regulator of mitochondrial biogenesis and energy metabolism, and it modulates chicken skeletal muscle development by regulating the flux of glycolysis and the tricarboxylic acid (TCA) cycle (Ma et al., 2022; Wang et al., 2024). *IGF2BP1* belongs to the insulin-like growth factor mRNA binding protein family, which mediates the insulin-like growth factor receptor signaling pathway to control embryonic development, postnatal growth, fat metabolism and adipogenesis (Chen et al., 2019; Guo et al., 2023).

### Novel candidate genes associated with ENs

Pleiotropic loci are of substantial utility in layer breeding programs, as single-marker selection enables simultaneous improvement of multiple genetically correlated traits. For egg number traits, we identified three novel pleiotropic loci, one in each of the A, H, and AH populations, with each locus associated with egg production across multiple stages of the laying cycle. In population A, the pleiotropic SNP resided in the intron of *ZNF592* on GGA10; in population H, the multi-trait locus was located in the intron of *DENND1B* on GGA8; and in the combined population AH, the shared pleiotropic variant mapped to the intron of *WDPCP* on GGA3.

*ZNF592* encodes a C2H2‑type zinc‑finger transcription factor. Transcriptomic evidence from mammals has shown that *ZNF592* is expressed in reproductive tissues including the ovary and testis, implying its potential roles in developmental regulation (Uhlén et al., 2015). *DENND1B* encodes a DENN domain containing guanine nucleotide exchange factor that activates RAB35 and mediates clathrin-dependent endocytosis and intracellular vesicular trafficking (Marat and McPherson, 2010). Mammalian transcriptomic profiles indicated that *DENND1B* is transcribed in reproductive tissues including ovary and pituitary (Uhlén et al., 2015). *WDPCP* participates in planar cell polarity and cilia formation, which maybe critical for ovarian follicle development and ovulation cycle regulation (Scott et al., 2013).

Notably, we observed very little overlap of significant SNPs for EN traits between populations A and H. This population‑specific pattern is consistent with their distinct genetic backgrounds and breeding goals, suggesting that egg number performance is under lineage‑dependent genetic regulation. When using the combined population AH for GWAS, we recovered extra loci absent from single‑line analyses. This supports that joint GWAS can boost statistical power to capture moderate‑effect variants segregating across the two populations.

### Candidate genes associated with MCD

Maximum consecutive egg laying days (MCD) reflects laying persistence and is an important indicator of egg production stability (Wang et al., 2022). In our study, MCD showed the highest coefficient of variation among all traits, suggesting more complex genetic regulation. Only one genome-wide significant SNP for MCD was detected, exclusively in populatin A, and it was located in the intron of *BMPR1A* on GGA6. At the suggestive level, population H harbored one candidate SNP in the intron of *MYCBP2* on GGA1.

*BMPR1A* functions as a key receptor of the bone morphogenetic protein signaling pathway, which plays established roles in regulating granulosa-cell proliferation, follicle selection, and atresia within the hen ovary (Onagbesan et al., 2003). Continuous egg‑laying performance requires well‑orchestrated follicular recruitment together with restrained follicular atresia. As such, *BMPR1A* is likely to affect MCD by modifying the dynamics of the ovarian follicular hierarchy. *MYCBP2* produces a MYC‑binding protein that modulates cell‑cycle progression and apoptosis. Transcriptomic evidence has highlighted MYC as an upstream regulator governing follicular recruitment in laying hens (Kramer et al., 2025). This supports the hypothesis that *MYCBP2* contributes to laying persistence through the regulation of ovarian cell survival. Given the population-specific nature of MCD associated genetic signals, laying persistence appears to be controlled by different regulatory mechanisms between the two breeds. For this reason, lineage-specific markers should be adopted for practical breeding targeting MCD.

### Complementary insights from selection signature and GWAS

No overlapping candidate genes were found between selection signature analysis (SSA) and GWAS, yet both sets partially overlapped with known egg production QTLs in the chicken QTLdb (*LSAMP, FARSB* from SSA; *NR3C2* from GWAS). This discrepancy is biologically interpretable: SSA detects regions under long-term directional selection where favorable alleles may be near fixation, lacking sufficient polymorphism for GWAS detection. In contrast, GWAS captures segregating variants driving individual phenotypic variation, which may not experience strong differential selection between breeds. Our results highlight the polygenic complexity of egg‑laying performance in chickens.

Functional enrichment analysis further supported the complementary roles of the two approaches. SSA candidate genes were mainly enriched in hormone response, nutrient level sensing, and growth hormone-related pathways, reflecting systemic metabolic and endocrine adaptation shaped by long-term selection for population level laying performance (Abdelmanova et al., 2021; Tan et al., 2024; Wang et al., 2024). The indigenous Jianghan chicken has undergone natural and artificial selection for stress tolerance, roughage resistance and meat‑egg quality, whereas Rhode Island Red has experienced long‑term intensive selection for high egg production. Genomic signatures in these pathways reflect reshaped trade‑offs between nutrition, metabolism and reproduction under artificial selection: commercial layers allocate more energy toward egg‑laying, while indigenous breeds maintain a balance among reproduction, survival and adaptability. GWAS candidate genes were enriched in calcium signaling, neuroactive ligand-receptor interaction, and apoptosis regulation, which directly influence follicular maturation, ovulation rhythm, and egg formation to produce individual differences in egg yield (Zhao et al., 2023; Shen et al., 2024; Sun et al., 2024; Du et al., 2025). Together, these findings imply that selection-driven loci establish the basal production capacity of the population, while segregating functional variants determine individual phenotypic variation.

## Conclusion

This study provides a joint GWAS of egg production traits in 490 Jianghan and 485 Rhode Island Red chickens. Our study systematically characterized the genetic architecture of egg production traits in these two genetically divergent layer populations. We identified a major shared WFE‑associated QTL on GGA4, harboring *LDB2* and *PPARGC1A*, and *IGF2BP1* on GGA27 was also a promising candidate for WFE. Three population‑specific pleiotropic loci for egg number traits contained *ZNF592, DENND1B*, and *WDPCP. BMPR1A* and *MYCBP2* were prioritized for MCD. Selection‑signature candidate genes were enriched in hormonal response and energy metabolism, while GWAS candidates related to follicular development, calcium signaling, and apoptosis regulation.These findings enrich our understanding of the genetic control of egg production traits, and the identified SNPs and candidate genes can serve as molecular markers to accelerate genomic selection breeding in laying hens.

## Authorship contributions

Ming Fu designed the research, performed data analysis and result interpretation, and drafted the original manuscript. Jinsong Pi, Yaru Chen, Ailuan Pan, Hao Zhang, and Tao Huang contributed to sample collection, experimental implementation, data acquisition, analysis, and interpretation. Yan Wu contributed to study design, result interpretation, and supervision of the research. All authors critically reviewed and revised the manuscript for important intellectual content, approved the final version to be published, and accept personal and public accountability for all aspects of this work. All authors ensure that any questions regarding the accuracy or integrity of the research will be thoroughly investigated and appropriately resolved.

## Ethics declaration

### Animal subject

This study was conducted in accordance with the ARRIVE (Animal Research: Reporting of In Vivo Experiments) guidelines. This study was approved by the Institute of Animal Science and Veterinary Medicine, Hubei Academy of Agricultural Sciences.

## Disclosures

No competing financial interests or personal connections that could bias the present study have been declared by the authors. The authors adopted Doubao AI to polish text readability during manuscript drafting. The authors performed manual review and modification of the AI-generated content and assume full responsibility for the published work.

## Footnotes

Supplementary material associated with this article can be found, in the online version, at doi:10.1016/j.psj.2026.107990.
